# Synthesis of Four Steroidal Carbamates with Antitumor Activity against Mouse Colon Carcinoma CT26WT Cells: In Vitro and In Silico Evidence

**DOI:** 10.3390/ijms23158775

**Published:** 2022-08-07

**Authors:** Daylin Fernández Pacheco, Dayana Alonso, Leonardo González Ceballos, Armando Zaldo Castro, Sheila Brown Roldán, Mairelys García Díaz, Anabel Villa Testa, Sarah Fuentes Wagner, Janet Piloto-Ferrer, Yamilet Coll García, Andrés F. Olea, Luis Espinoza

**Affiliations:** 1 Center for Natural Product Research, Faculty of Chemistry, University of Havana, Zapata and G, Havana 10400, Cuba; 2 Laboratory of Synthetic and Biomolecular Chemistry, Faculty of Chemistry, University of Havana, Havana 10400, Cuba; 3 Antidoping Laboratory, Havana 10800, Cuba; 4 Drug Research and Development Center (CIDEM), Havana 10400, Cuba; 5 Grupo QBAB, Instituto de Ciencias Químicas Aplicadas, Facultad de Ingeniería, Universidad Autónoma de Chile, Llano Subercaseaux 2801, San Miguel, Santiago 7500912, Chile; 6 Departamento de Química, Universidad Técnica Federico Santa María, Avenida España 1680, Valparaíso 2340000, Chile

**Keywords:** synthesis, steroidal carbamates, colorectal cancer, molecular docking

## Abstract

Colorectal cancer (CRC) is one of the most lethal cancers worldwide. If detected on time, surgery can expand life expectations of patients up to five more years. However, if metastasis has grown deliberately, the use of chemotherapy can play a crucial role in CRC control. Moreover, the lack of selectivity of current anticancer drugs, plus mutations that occur in cancerous cells, demands the development of new chemotherapeutic agents. Several steroids have shown their potentiality as anticancer agents, while some other compounds, such as Taxol and its derivatives bearing a carbamate functionality, have reached the market. In this article, the synthesis, characterization, and antiproliferative activity of four steroidal carbamates on mouse colon carcinoma CT26WT cells are described. Carbamate synthesis occurred via direct reaction between diosgenin, its B-ring modified derivative, and testosterone with phenyl isocyanate under a Brønsted acid catalysis. All obtained compounds were characterized by ^1^H and ^13^C Nuclear Magnetic Resonance (NMR), High Resolution Mass Spectroscopy (HRMS); their melting points are also reported. Results obtained from antiproliferative activity assays indicated that carbamates compounds have inhibitory effects on the growth of this colon cancer cell line. A molecular docking study carried out on Human Prostaglandin E Receptor (EP4) showed a high affinity between carbamates and protein, thus providing a valuable theoretical explanation of the in vitro results.

## 1. Introduction

Colorectal cancer (CRC) is one of the most widespread (worldwide) and lethal cancers reported to date. It is the third most commonly occurring cancer in men and the second most commonly occurring cancer in women. In 2018, the number of new CRC cases surpassed 1.8 million, while deaths reached 0.9 million. It is estimated that this year, 2 million new cases will be diagnosed all over the world, with an estimate of nearly 1 million deaths. Early detection of CRC can provide a 5-year survival rate of up to 90%, and surgery is most often curative. However, if patients carry a distant metastasis at the time of diagnosis, the 5-year survival rate drops to only 10% [1]. Almost half of patients with colon cancer will relapse, highlighting the need for improvement in current treatment regimens. To investigate biological mechanisms underlying colon cancer progression and to design more effective drugs, it is essential to develop effective therapies against CRC.

Mouse colon carcinoma CT26WT cells have been used as a model for the evaluation of immunotherapy protocols in various immune response studies [2]. Furthermore, they are frequently used as a model to investigate antitumor drug–drug interactions in combination therapy schemes [3] and in the evaluation of new antitumor candidates. Used in over 500 published studies, CT26WT colon carcinoma cells are one of the most employed cell lines in drug development [4,5].

**Figure 1:**
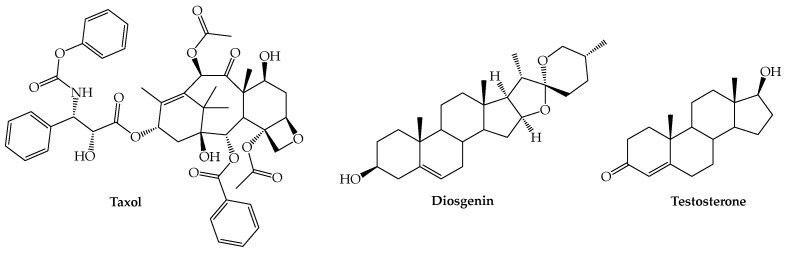
Structures of Taxol, Diosgenin, and Testosterone.

Current anticancer drugs have several drawbacks, mainly a lack of selectivity towards tumor cells [6]. On the other hand, cancer cells mutate, developing resistance towards chemotherapeutic agents [7]. Thus, the need for new anticancer drugs is urgent, and therefore the quest for novel agents is a very active research field. For many decades, natural products have been used directly, such as Taxol (Figure 1), or as templates to synthesize analogs, with anticancer activities depending on their chemical structures [8,9,10]. In this context, steroids emerge as an attractive alternative since they are essential components in cell membranes developing several physiological functions [11,12]. Lipophilic steroids, such as diosgenin and testosterone (Figure 1), can easily enter most cells and interact with intracellular receptors; they have been used as scaffold for synthesis of anticancer agents against a wide range of cancer forms, including multi-drug-resistant cancers [13]. In the case of diosgenin, several publications have reported its antitumor properties and, more specifically, its ability to induce growth inhibition in different colon cancer cells [14,15,16,17]. On the other hand, testosterone, and its derivatives, are known as in vitro inhibitors of mammary and prostate cancers [18,19,20,21,22].

**Figure 2:**
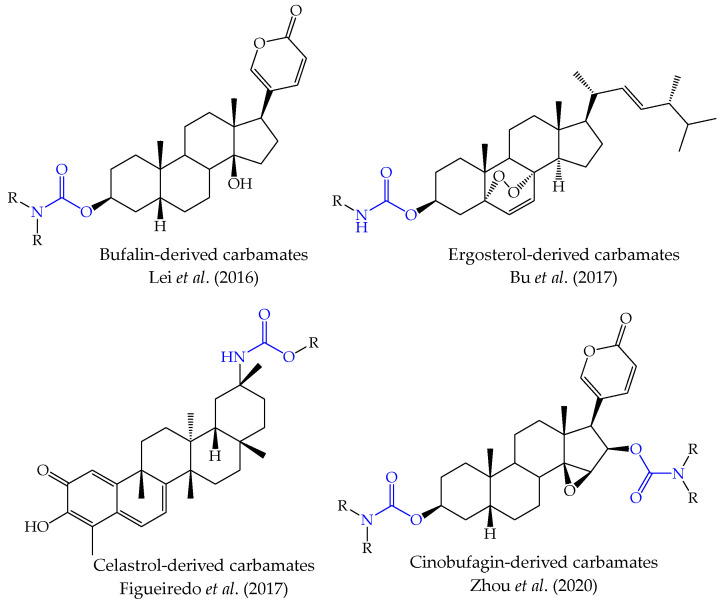
Recently reported in vitro and in vivo active steroidal carbamates [24,25,26,27].

In addition to Taxol, which is a widely used anticancer drug [23], a series of steroidal carbamates have recently been synthesized, and their anticancer, cytotoxic, and antitumor properties have been evaluated [24,25,26,27] (Figure 2).

However, to the best of our knowledge, no studies on diosgenin- or testosterone-carbamate derivatives have been published to date.

In the case of CRC, it is well established that colon carcinoma cells CT26 produce prostaglandin E_2_ (PGE_2_), which induces cancer cell growth, migration, metastasis via stimulation of the PGE_2_/EP4 receptor, and activation of extracellular signal-regulated kinase (ERK) [28]. Recently, it has been proposed that the blockade of this receptor can be used to ameliorate several human diseases, including cancer effects [29], and more specific targeting of EP4 has been considered as a promising therapeutic approach for CRC therapy [30,31]. Targeting receptors as cancer therapy is under extensive research, and natural products have been tested as ligands for many receptors involved in cancer development [32].

Thus, the synthesis of four steroidal carbamates, three derived from diosgenin and one from testosterone, and the assessment of their cytotoxic activity in mouse colon carcinoma CT26WT cells is described. In addition, a molecular docking study between these carbamates and the Human Prostaglandin E Receptor (EP4) has been performed. Theoretical results are used to explain experimental in vitro activities.

## 2. Results and Discussion

### 2.1. Synthesis of Steroidal Carbamates

**Figure 3:**
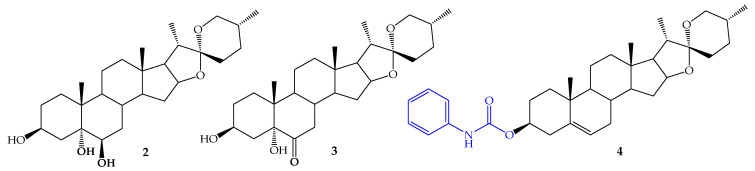
Diosgenin derivatives **2** and **3,** and steroidal carbamate **4**.

Synthesis of diosgenin derivatives **2** and **3** (Figure 3) have been previously reported [33]. Briefly, a diosgenin double bond is epoxidized, followed by enantioselective opening of the obtained epoxides, and regioselective oxidation of the axial -OH group at C6. On the other hand, synthesis of carbamate **4** was performed by reacting diosgenin with phenyl isocyanate under Brønsted acid catalysis.

**Scheme 1:**
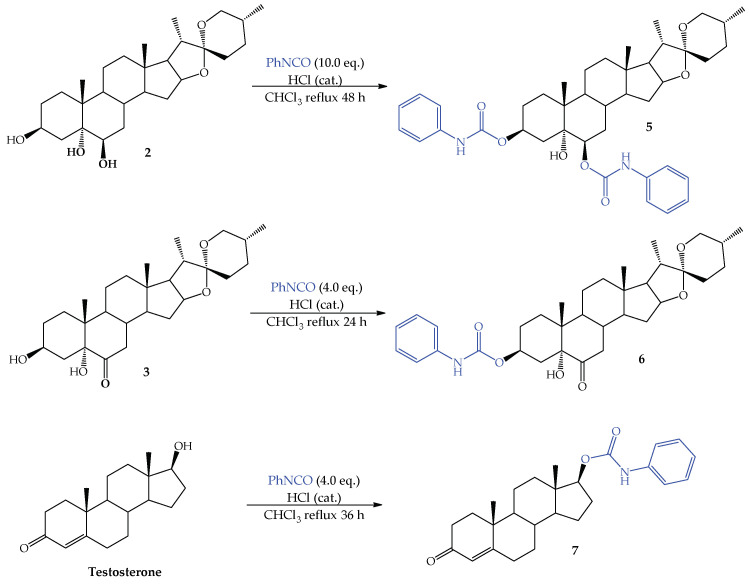
Synthesis of diosgenin carbamates **5** and **6**, and testosterone carbamate **7**.

Using this reaction, three new carbamates were synthesized, namely diosgenin carbamates **5** and **6**, and testosterone carbamate **7** (Scheme 1). Conditions for condensation reactions between hydroxysteroids and phenyl isocyanate under hydrochloric acid catalysis are shown in Scheme 1.

All condensations were carried out in a chloroform solution at reflux temperature and monitored by thin-layer chromatography (TLC) for 24–48 h. The pure compounds **5**, **6,** and **7** were separated from crude reaction mixtures by column chromatography with moderate yields (55–68%). NMR spectra of compounds 5, 6 and 7 are given in Figure S1, Figure S2 and Figure S3, respectively.

### 2.2. Antiproliferative Activity of Compounds **4**, **5**, **6,** and **7** on CT26WT Cells

**Figure 4:**
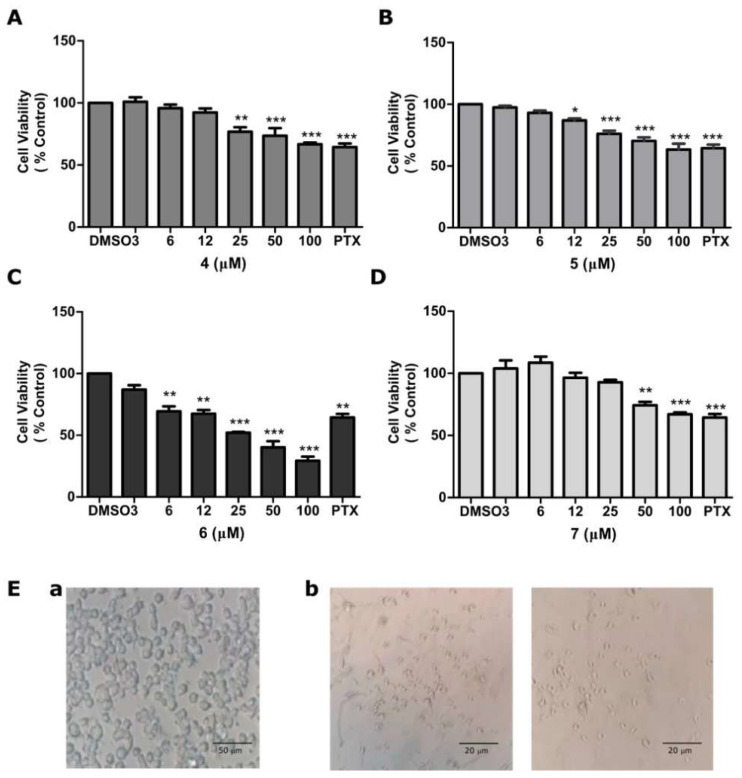
Antiproliferative effects of **4**–**7** on CT26WT cells. Cells at the mid-log phase were seeded in a 96-well plate at 1 × 10^4^ cells density in 100 µL medium. After being cultured overnight, cells were exposed to compounds **4**, **5**, **6, 7** (3, 6, 12, 25, 50 and 100 µM) and Placlitaxel (PTX) (50 µM) for 24 h. The cells were incubated with MTT (5 mg/mL) for 4 h. Inhibitory effects of 4 (**A**), 5 (**B**), 6 (**C**) and 7 (**D**) in cell viability of CT26WT cells were determined by the MTT assay. Data are presented as mean ± SD (n = 3), results of an ANOVA, and the Dunnet Test. Significance: * *p* < 0.05, ** *p* < 0.01, *** *p* < 0.001 vs. negative control (DMSO). (**E**) Representative pictures: (**a**) untreated cells, (**b**) morphological changes induced by compound **6** at 50 µM.

Cytotoxic effects of compounds **4**, **5**, **6**, and **7** on CT26WT cells were evaluated by assessing the alterations in cell morphology, and subsequently by measuring the 3-(4,5-dimethylthiazol-2-yl)-2,5-diphenyltetrazolium bromide (MTT) reduction. These assays were used to determine the antiproliferative effects of carbamates on CT26WT cell growth after 24 h of treatment. Results shown in Figure 4 indicate that all steroids exhibit inhibitory effects on CT26WT cell growth in a concentration-dependent manner, and all differences are statistically significant compared to untreated controls (*p* < 0.05).

Initial inhibitory effects of compounds **4**, **5**, **6,** and **7** are observed at 25 μM, 12 μM, 6 μM, and 50 μM, respectively (Figure 4A–D). A comparison of cell viability in the presence of carbamates at 25 μM indicates that the inhibitory activity follows the order: **7** < **4** = **5** < **6**. For the most active carbamate (**6**), an IC_50_ value of 26.8 μM was obtained, which is in the same order of previously reported values in the literature for steroidal carbamates derivatives [34,35].

On the other hand, morphological changes such as cell shrinkage, roundup, and extensive cell detachment from the culture substratum were also observed, becoming progressively visible with increasing concentrations of compounds **4**–**7**, but were absent in control cells (Figure 4Ea,b). 

### 2.3. Molecular Docking Study

Redocking experiments were carried out using the co-crystallized form of EP4 complexed with ONO-AE3-208, an EP4 antagonist. Only slight structural differences were found between the docked structure and its conformation in the experimental complex [36]. In these preliminary docking simulations, a low RMSD value (2.99 Å) was found, suggesting the chosen parameters, such as location and size of the simulation box, were suitable.

Docking results obtained for **4**, **5**, **6,** and **7** indicate that these compounds bind to EP4 with negative binding energies and a relatively large population of their clusters (Table 1). According to Rosenfeld criteria [37], a binder will present low binding energy and a few (max 2) different binding modes. In the case of compounds **4**, **5,** and **6,** a small number of different conformations with high population were obtained. On the other hand, four clusters were found for **7**, which is in good agreement with experimental results, suggesting that this compound is the weakest growth inhibitor of CT26WT cell line. Despite these outcomes, the energy obtained via docking experiments should be treated carefully because it depends on several factors, such as the number and type of atoms and the number of rotatable bonds of the tested ligand. This is the reason why contact-based analysis is useful for understanding the biological behavior of analyzed compounds.

**Figure 5:**
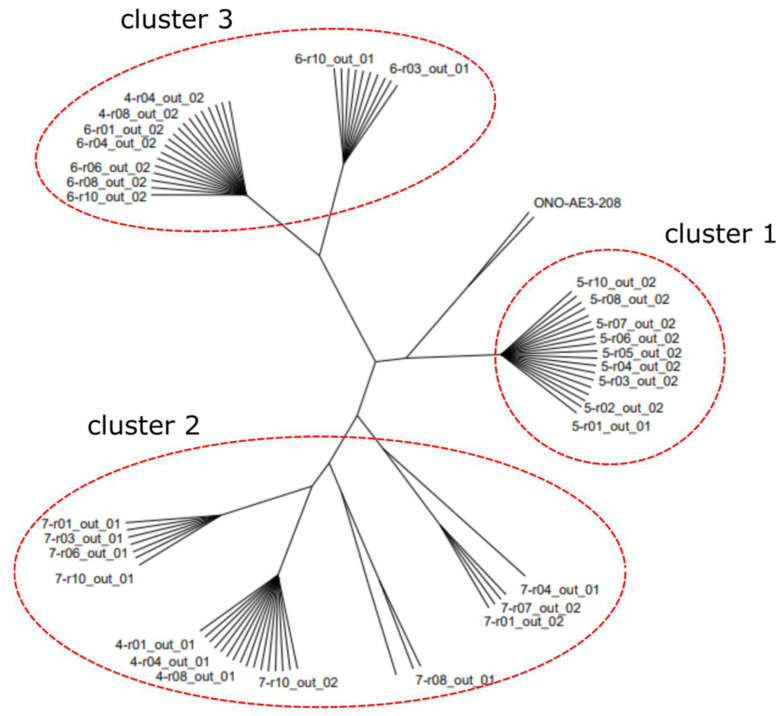
Map tree obtained from AuPosSOM. Each cluster represents a different ligand footprint on the EP4 ligand complex. The co-crystalized inhibitor of EP4 is shown as ligand.

Afterwards, docking results were evaluated by performing automatic analysis of poses using self-organizing maps (AuPosSOM). Ligands are clustered by considering the similarity of their poses contact footprint with the EP4 receptor, and a tree map showing contact footprint of all poses was obtained (Figure 5). For the sake of comparison, the footprint of a co-crystallized inhibitor (ONO-AE3-208) has also been included. As displayed in Figure 5, three major clusters were obtained, and the highest similarity with the ONO-AE3-208 binding mode corresponds to compound **5** (Cluster 1. Figure 5). Only binding modes 1 and 3 of compound **7** were considered for further analysis.

A contact-based analysis using BINANA was performed to obtain an insight into the kind and strength of ligand–receptor interactions (Table S1). As a general trend, only a few hydrogen bonds were found among the predicted conformations (Figure S4). Compounds **4** (2) and **7** (1) exhibited polar interactions with T76 at 2.4 and 2.5 Å, respectively, while **6** (1) formed a hydrogen bond (1.9 Å) with Y80. Interestingly, a polar interaction between R316 and the hydroxyl group bound to C5 on the steroidal core of compound **7** (2) was also observed. On the other hand, several van der Waals contacts (<4.0 Å) were identified, with the most recurrent involving T76, Y80, R316, and V320; the first three are crucial residues for ligand binding [27].

**Figure 6:**
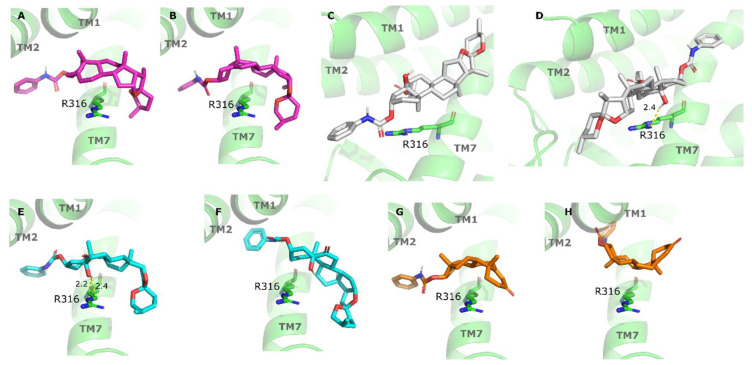
Binding modes of (**A**) **4** (1), (**B**) **4** (2), (**C**) **5** (1), (**D**) **5** (2), (**E**) **6** (1), (**F**) **6** (2), (**G**) **7** (1), and (**H**) **7** (3) within the active site of EP4. EP4 is displayed similar to a green cartoon and R316 residue is shown as green sticks. All distances are given in Å.

Binding modes of **4**, **5**, **6,** and **7**, are shown in Figure 6. Interestingly, in all poses except for **5** (2), the N-phenyl-carbamoyl moiety was oriented towards residues of the second transmembrane domain (TM2), while the steroid moiety was located between TM1 and TM7 with the α-face of steroidal rings near to TM7 (Figure 6A). On the other hand, in pose **5,** (2) the N-phenyl-carbamoyl fragments were oriented towards TM1 and TM7 and the steroidal moiety was also placed towards TM2 (Figure 6D). It is remarkable that only **6** (1) kept two short-distance interactions (<2.5 Å) with carbons at γ and δ positions of R316 (Figure 6E,F), whereas **5** (2) formed a polar interaction with guanidine group of R316 at 2.4 Å. These interactions involved the proton from the 5-hydroxyl group, suggesting B-ring functionalization with polar groups could be a positive feature in establishing strong interactions with EP4. These results indicate that compound **6** should be the best EP4 antagonist, which is in line with the experimental data presented here.

**Figure 7:**
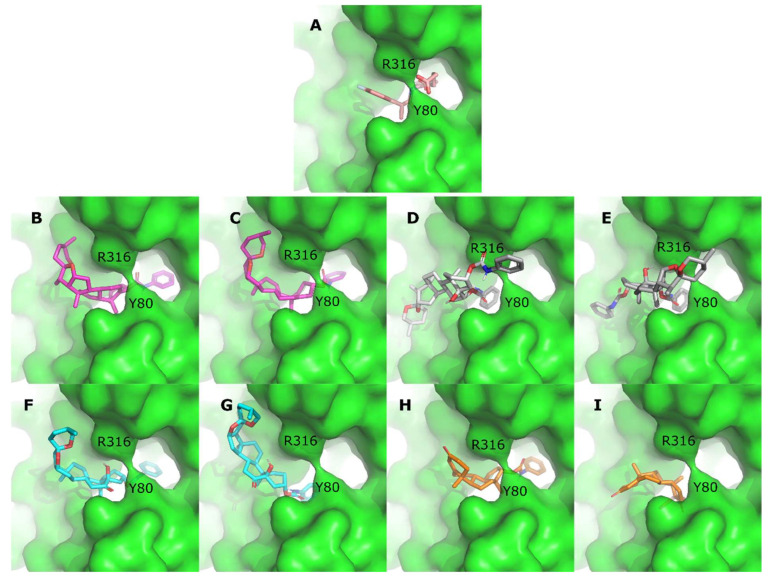
Binding modes of (**A**) ONO-AE3-208, (**B**) **4** (1), (**C**) **4** (2), (**D**) **5** (1), (**E**) **5** (2), (**F**) **6** (1), (**G**) **6** (2), (**H**) **7** (1), and (**I**) **7** (3) within the active site of EP4.

Theoretical volume values for compounds **4**: 523.14, **5**: 648.86, **6**: 539.23, and **7**: 395.30 Å^3^ were determined by Molinspiration [39]. Interestingly, binding modes obtained for compounds with similar volumes such as **4** (Figure 7B,C) and **6** (Figure 7F), show that the N-phenyl-carbamoyl fragment was located inside the cavity, similar to the co-crystalized inhibitor (Figure 7A) where the steroidal moiety was blocking the active site entrance. Thus, the lower antiproliferative activity of compound **5** could be attributed to its higher molecular volume given by the two phenyl-carbamoyl groups. This structure does not fit into the receptor cavity (Figure 7D,E) because of steric hindrance, and therefore gives a lower number of common interactions with the control compound. On the other hand, compound **7**, which had the lowest molecular volume, was inside the receptor cavity (Figure 7H) and four different binding modes were found. These results suggest that molecules with large or small volumes are not suitable for obtaining good modes of interaction with the receptor.

## 3. Materials and Methods

### 3.1. General

All chemical reagents were purchased from Merck (Darmstadt, Germany), Fluka (Darmstadt, Germany), or Sigma-Aldrich (St. Louis, MI, USA) and used without previous purification. All solvents were distilled and stored over proper desiccants. Melting points were measured on a BUCHI M-565 equipment. NMR spectra were recorded at 298 K on 400 NMR spectrometers, Varian Mercury (Varian, Palo Alto, CA, USA) and Avance Neo 400 Digital (Bruker, Rheinstetten, Germany), at 400 MHz and 101 MHz for ^1^H and ^13^C, respectively. All spectra were referenced using the TMS signal or the residual peak of the solvent. Chemical shifts (δ) are reported in ppm and coupling constants (J) are given in Hz. A TripleToF 6600-1 (Sciex, MA, USA) mass spectrometer was used for high-resolution mass spectrometry (HRMS). Silica gel (Merck, Darmstadt, Germany, 70–230 mesh) was used for column chromatography and silica gel plates (Fluka, Darmstadt, Germany), and HF_254_ for thin-layer chromatography (TLC). TLC spots were detected by heating after staining with cerium molybdate in H_2_SO_4_. The cell viability analysis was conducted on a microplate spectrophotometer (OMEGA) at 570 nm. All abbreviation used are listed in Table S2.

### 3.2. Methods of Synthesis

#### 3.2.1. Synthesis of (25R)-5α-Hydroxy-spirostan-3β,6β-yl Phenylcarbamate (**5**)

To a solution of compound **2** (1.0 g, 2.2 mmol) in CHCl_3_ (50 mL), phenyl isocyanate (2.44 mL, 22.3 mmol) and some drops of HCl (37% *w*/*w* in water) were added. The reaction was refluxed for 48 h and followed by TLC (n-Hex/EtOAc 3:1). Next, the obtained solution was concentrated to dryness. The crude product was purified by column chromatography (n-Hex/EtOAc 3:1) and compound **5** was obtained (0.84 g, 55% yield). Compound **5** white solid, m.p. 166–168 °C, Rf = 0.44 (CH_2_Cl_2_/EtOAc 6:1). ^1^H NMR (400 MHz, CDCl_3_) (Figure S1) δ 7.40 (d, *J* = 8.0 Hz, 2H, H-2a), 7.36–7.23 (m, 6H, H-3a, H-2b, H-3b), 7.07 (t, *J* = 7.2 Hz, 1H, H-4a), 7.03 (tt, *J* = 7.2 and 1.4 Hz, 1H, H-4b), 6.54 (s, 2H, NH), 5.30 (s, 1H, C5-OH), 5.15 (tt, *J* = 11.0 and 5.4 Hz, 1H, H-3), 4.76 (d, *J* = 3.0 Hz, 1H, H-6), 4.39 (td, *J* = 8.4, 8.0 and 4.3 Hz, 1H, H-16), 3.51–3.43 (m, 1H, H-26 eq), 3.37 (t, *J* = 10.9 Hz, 1H, H-26 ax), 2.03–1.93 (m, 3H), 1.90–1.84 (m, 2H), 1.84–1.78 (m, 2H), 1.78–1.68 (m, 2H), 1.68 (s, 3H, H-19), 1.67–1.54 (m, 4H), 1.52–1.38 (m, 4H), 1.38–1.09 (m, 7H), 0.97 (d, *J* = 6.9 Hz, 3H, H-21), 0.80 (s, 3H, H-18), 0.78 (d, *J* = 6.3 Hz, 3H, H-27). ^13^C NMR (101 MHz, CDCl_3_) (Figure S1) δ 153.06 (NHCO-29), 152.74 (NHCO-30), 137.87 (C-1b), 137.68 (C-1a), 129.08 (C-3b), 129.00 (C-3a), 123.65 (C-4b), 123.33 (C-4a), 118.63 (C-2b and C-2a), 109.20 (C-22), 80.71 (C-16), 76.83 (C-6), 75.01 (C-5), 71.58 (C-3), 66.82 (C-26), 62.06 (C-17), 55.55 (C-14), 44.98 (C-9), 41.64 (C-20), 40.67 (C-13), 39.88 (C-12), 38.57 (C-10), 37.17 (C-4), 31.79 (C-1 and C-7), 31.65 (C-15), 31.38 (C-23), 30.44 (C-8), 30.29 (C-25), 28.79 (C-24), 26.97 (C-2), 20.86 (C-11), 17.11 (C-27), 16.66 (C-18), 16.59 (C-19), 14.47 (C-21). ESI-HRMS, calculated for C_41_H_55_N_2_O_7_: 687.4009 [M + H]^+^ found: *m*/*z* 687.4047.

#### 3.2.2. Synthesis of (25R)-5α-Hydroxy-6-oxo-spirostan-3β-yl Phenylcarbamate (**6**)

To a solution of compound **3** (1.0 g, 2.2 mmol) in CHCl_3_ (50 mL), phenyl isocyanate (0.96 mL 1.12 mL, 8.8 mmol) and some drops of HCl (37% *w*/*w* in water) were added. The reaction was refluxed for 24 h and followed by TLC (n-Hex/EtOAc 3:1). Next, the obtained solution was concentrated to dryness. The crude product was purified via column chromatography (n-hex/EtOAc 3:1) and compound **6** was obtained (0.86 g, 68% yield). Compound **6** white solid, m.p. 264–266 °C, Rf = 0.32 (n-hex/EtOAc 3:1). ^1^H NMR (400 MHz, CDCl_3_) (Figure S2) δ 7.32 (d, *J* = 7.7 Hz, 2H, H-2a), 7.29 (d, *J* = 7.1 Hz, 2H, H-3a), 7.09–7.00 (m, 1H, H-4a), 6.60 (s, 1H, NH), 5.04 (tt, *J* = 10.7 and 5.0 Hz, 1H, H-3), 4.41 (q, *J* = 7.8 and 6.3 Hz, 1H, H-16), 3.47 (ddd, *J* = 11.0, 4.6 and 1.9 Hz, 1H, H-26 eq), 3.36 (t, *J* = 10.9 Hz, 1H, H-26 ax), 2.90 (s, 1H, C5-OH), 2.81–2.68 (m, 1H, H-7 ax), 0.97 (d, *J* = 6.8 Hz, 3H, H-27), 0.84 (s, 3H, H-19), 0.79 (d, *J* = 6.3 Hz, 3H, H-21), 0.75 (s, 3H, H-18). ^13^C NMR (101 MHz, CDCl_3_) (Figure S2) δ 211.98 (C-6), 153.39 (NHCO), 137.83 (C-1a), 129.19 (C-3a), 123.69 (C-4a), 119.00 (C-2a), 109.42 (C-22), 80.64 (C-5), 80.48 (C-16), 71.70 (C-3), 67.00 (C-26), 62.18 (C-17), 56.20 (C-14), 44.48 (C-9), 42.65 (C-10), 41.96 (C-20) 41.76 (C-7), 41.22 (C-13), 39.71 (C-12), 36.92 (C-4), 32.96 (C-8), 31.70 (C-15), 31.49 (C-23), 30.42 (C-1), 29.70 (C-25), 28.93 (C-24), 26.76 (C-2), 21.37 (C-11), 17.27 (C-19), 16.56 (C-27), 14.59 (C-18), 14.18 (C-21). ESI-HRMS, calculated for C_34_H_48_NO_6_: 566.3481 [M + H]^+^ found: *m*/*z* 566.3527.

#### 3.2.3. Synthesis of 4-En-androst-17β-yl Phenylcarbamate (**7**)

To a solution of testosterone (1.0 g, 3.5 mmol) in CHCl_3_ (30 mL), phenyl isocyanate (1.5 mL, 14 mmol) was added dropwise along with some drops of HCl (37% *w*/*w* in water). The reaction mixture was refluxed for 36 h and followed by TLC (n-hex/EtOAc 5:1). Next, the obtained solution was concentrated to dryness. The crude product was purified via column chromatography (n-Hex/EtOAc 5:1) and compound **7** was obtained (0.95 g, 67% yield). Compound **7** white solid, m.p. 290–291 °C. Rf = 0.53 (n-hex/EtOAc 5:1). ^1^H NMR (400 MHz, CDCl_3_/DMSO-d_6_ 6:1) (Figure S3) δ 7.78 (s, 1H, NH), 7.28 (d, *J* = 7.8 Hz, 2H, H-2a), 7.16–7.07 (m, 2H, H-3a), 6.90–6.81 (m, 1H, H-4a), 5.56 (d, *J* = 1.7 Hz, 1H, H-4), 4.49 (t, *J* = 8.5 Hz, 1H, H-17), 2.33–2.19 (m, 2H), 2.24–2.10 (m, 2H), 2.06 (ddt, *J* = 15.7, 9.3 and 5.0 Hz, 1H), 1.88 (ddd, *J* = 13.4, 5.0 and 3.2 Hz, 1H), 1.77–1.66 (m, 2H), 1.62–1.49 (m, 2H), 1.51–1.38 (m, 2H), 1.34–1.23 (m, 1H), 1.23–1.14 (m, 1H), 1.19–0.98 (m, 2H), 1.04 (s, 3H, H-19), 0.99–0.92 (m, 1H), 0.92–0.86 (m, 1H), 0.81 (ddd, *J* = 12.2, 10.7 and 4.0 Hz, 1H), 0.73 (s, 3H, H-18). ^13^C NMR (101 MHz, CDCl_3_/DMSO-d_6_ 6:1) (Figure S3) δ 199.28 (C-3), 171.11 (C-5), 153.69 (NHCO), 138.40 (C-1a), 128.57 (c-3a), 123.54 (C-4), 122.58 (C-4a), 118.36 (C-2a), 82.68 (C-17), 53.43 (C-9), 49.91 (C-14), 42.25 (C-13), 38.38 (C-10), 36.43 (C-12), 35.38 (C-1), 35.14 (C-8), 33.66 (C-2), 32.49 (C-6), 31.19 (C-7), 27.39 (C-15), 23.12 (C-16), 20.28 (C-11), 17.13 (C-19), 11.92 (C-18). ESI-HRMS, calculated for C_26_H_34_NO_3_: 408.2538 [M + H]^+^ found: *m*/*z* 408.2555.

### 3.3. Anticancer Studies

#### 3.3.1. Cell Line and Culture Condition

Mouse colon carcinoma CT26WT cells were acquired from the Immunology Molecular Center, La Habana, Cuba. Cell cultures were grown in RPMI 1640 medium containing 10% fetal bovine serum (FBS), 100 IU/mL penicillin, and 100 µg/mL streptomycin. Cells were kept in a humidified environment at 37 °C and an air atmosphere of 5% CO_2_. For harvesting, cells were trypsinized using standard procedures.

#### 3.3.2. Cell Viability Analysis

The MTT [3-(4,5-dimethylthiazol-2-yl)-2,5-diphenyltetrazolium bromide] assay was used to assess the effect of carbamates on the viability of CT26WT cells [40]. Briefly, cells at the mid-log phase were seeded in a 96-well plate at 1 × 10^4^ cells density in 100 µL medium. After being cultured overnight, cells were exposed to compounds **4**, **5**, **6,** and **7** (3, 6, 12, 25, 50, and 100 µM), and Paclitaxel (Taxol^®^) (50 µM). At the end of this period, cells were incubated with MTT (Sigma, St. Louis, MO, USA) (5 mg/mL) for 4 h. The plates were read in a microplate spectrophotometer (OMEGA) at 570 nm. The IC_50_ was determined by fitting the viability percentage against concentration to a dose–response curve by using six different concentrations. The percentage of viable cells was calculated as the relative optical density compared to the control.

$$\mathrm{viability}\;\left(\%\right)=\frac{\mathrm{OD}\;\mathrm{values}\;\mathrm{of}\;\mathrm{treated}\;\mathrm{samples}}{\mathrm{OD}\;\mathrm{values}\;\mathrm{of}\;\mathrm{non}-\mathrm{treated}\;\mathrm{samples}}\times 100$$

### 3.4. Molecular Docking

The inhibition capacity of synthesized compounds to Human Prostaglandin E Receptor EP4 (EP4) was predicted using molecular docking. The EP4 3D structure resolved by X-ray diffraction PDB code: 5YWY resolution (R = 3.2 Å) [41] was downloaded from Protein Data Bank (http://www.rcsb.org) The inhibitor ONO-AE3-208 that is the co-crystalized ligand in 5YWY structure was used as the control compound. The 2D and 3D structures of the synthetic ligands were obtained with ChemBioDraw Ultra 14.0 and optimized with MOPAC 2016 (http://openmopac.net/) using AM1 semi-empirical Hamiltonian. Protein and ligands PDB files were converted to PDBQT format using AutoDockTools. Partial charges were calculated using the Gasteiger model. Nonpolar hydrogen atoms were merged to the heavy atoms. In the case of ligands, rotatable bonds were set to default using the TORSDOF utility in AutoDockTools [42]. All protein residues were kept rigid. A simulation box of size 24 Å × 22 Å × 24 Å was built [38]. The center of the simulation box was placed at the center of the active site.

Multiple rigid molecular docking simulations were performed using AutoDock Vina 1.1.2 program (Vina) [38]. The docking parameters were set to default except for the following: exhaustiveness = 32 and num_modes = 2. Then, 10 independent runs were carried out. The Vina-predicted enzyme–ligand complexes (20 docked poses per ligand) were clustered using AuPosSOM [43,44]. This program grouped the ligands considering the poses’ contact footprint with EP4 in comparison with ONO-AE3-208. The mean binding energy (kcal/mol) was determined for each cluster. Then, a contact-based analysis of the best-scoring pose in each group was carried out. Noncovalent interactions within ligands and EP4 were determined using the Python-implemented computer algorithm BINANA (Table S1) [45]. The binding modes of each ligand were represented using PyMOL 2.4.1. (Figure S4).

## 4. Conclusions

The synthesis and full characterization of steroidal carbamates obtained from diosgenin, including two of its derivatives, and testosterone have been described. The antiproliferative effects of these compounds on mouse colon carcinoma CT26WT cells were evaluated. Preliminary results show that all four carbamates possess antiproliferative activity in a concentration-dependent manner (6 to 50 uM). Compound **6** turned out to be the most active of this series, with a measured IC_50_ value equal to 26.8 uM, which is in the same order of magnitude of those previously reported for steroidal carbamate derivatives with anticancer properties. As a complement to this research, a molecular docking study was performed using carbamates **4**, **5**, **6,** and **7** as ligands and EP4 protein as receptor. The obtained results show that all tested compounds exhibit a high level of affinity towards this receptor. Analysis of poses and binding energies suggests that introduction of hydroxyl and carbonyl substituents at position 5 and 6 in ring B of the steroidal core could enhance antiproliferative activity via inhibition of the EP4 receptor. In summary, our results indicate diosgenin and testosterone carbamates exhibit antiproliferative activity against CRC, mainly via inhibition of the EP4 receptor. Additionally, this activity is determined by the nature of substituent on the steroid chemical structure, and therefore these steroids’ carbamates can be used as a scaffold for developing potential anticancer agents.

## Figures and Tables

**Table 1:** Binding energy and population of **4**, **5**, **6,** and **7** into EP4 using AutoDock Vina [38].

Ligands	Binding Mode	Poses Number	Energy (kcal/mol)
**4**	1	10	−9.76
	2	10	−8.60
**5**	1	10	−7.43
	2	9	−7.51
**6**	1	10	−8.29
	2	9	−8.10
**7**	1	10	−8.90
	2	3	−8.46
	3	4	−8.52
	4	3	−8.50

## Data Availability

Not applicable.

## Supplementary Materials

The supporting information can be downloaded at: https://www.mdpi.com/article/10.3390/ijms23158775/s1.
