# Supplementary material for: Synthesis of Four Steroidal Carbamates with Antitumor Activity against Mouse Colon Carcinoma CT26WT Cells: In Vitro and In Silico Evidence
Source: Int J Mol Sci. 2022 Aug 7;23(15):8775. doi: 10.3390/ijms23158775 (PMC9369283; doi:10.3390/ijms23158775)
Supplement: Supplementary file 1 [file ijms-23-08775-s001.zip › ijms-1841758-supplementary.pdf]

Type of the Paper (Article)

Synthesis of four steroidal carbamates with antitumor activity against mouse colon carcinoma CT26WT cells: *in vitro* and *in silico* evidence

Daylin Fernández Pacheco[1], Dayana Alonso[2], Leonardo González Ceballos[2], Armando Zaldo Castro[1], Sheila Brown Roldán[3], Mairelys García Díaz[1], Anabel Villa Testa[4], Sarah Fuentes Wagner[4], Janet Piloto-Ferrer[4], Yamilet Coll García[1]*, Andrés F. Olea[5], Luis Espinoza[6]*.

[1] Center for Natural Product Research, Faculty of Chemistry, University of Havana, Zapata and G, Havana 10400, Cuba; daylinfp@fq.uh.cu (D.F.P.); zaldo@fq.uh.cu (A.Z.C.); 9802mgd@gmail.com (M.G.D); yamcoll@fq.uh.cu (Y.C.G.).
[2] Laboratory of Synthetic and Biomolecular Chemistry, Faculty of Chemistry, University of Havana, Havana 10400, Cuba; dayana.alonso@fq.uh.cu (D. A); leo@fq.uh.cu (L.G.C.).
[3] Calle 100, esquina Aldabó. CP. 10800 Rancho Boyeros, La Habana, Cuba; sbrownr1594@gmail.com (S.B.R).
[4] Drug Research and Development Center (CIDEM), Avenida 26, No. 1605 e/ Puentes Grandes and Boyeros, Havana, Cuba; janet.piloto@cidem.cu (J. P-F.), anabel.villa@cidem.cu (A.V.T), sarah.fuentes@cidem.cu (S.F.W).
[5] Grupo QBAB, Instituto de Ciencias Químicas Aplicadas, Facultad de Ingeniería, Universidad Autónoma de Chile, Llano Subercaseaux 2801, San Miguel, Santiago, Chile; andres.olea@uautonoma.cl (A.O).
[6] Departamento de Química, Universidad Técnica Federico Santa María, Avenida España 1680, Valparaíso 2340000, Chile; luis.espinozac@usm.cl (L.E.)
* Correspondence: yamcoll@fq.uh.cu (Y.C.G.), Tel.: +53-52952050, Tel.: +53-52127844; luis.espinozac@usm.cl (LE), Tel.: +56-32-2654225.

Supplementary Materials: **Figure S1**: NMR spectra of (25R)-5α-hydroxy-Spirostan--3β, 6 β-yl phenylcarbamate (**5**); **Figure S2**: NMR spectra of (25R)- 5α-hydroxy-6-oxo-spirostan-3β-yl phenylcarbamate (**6**); **Figure S3**: NMR spectra of 4-en-androst-17β-yl phenylcarbamate (**7**); **Table S1:** Receptor-ligand interactions found by BINANA; **Figure S4:** Interaction pattern of A) **4**(1), B) **4**(2), C) **5**(1), D) **5**(2) E) **6**(1), F) **6**(2), G) **7**(1) and H) **7**(3) within the active site of EP4. Hydrogen bonds are highlighted with yellow dashed lines. Residues with van der Waals interactions are displayed as green sticks. All distances are given in Å. **Table S2:** Abbreviations used in this work.

Figure S1: NMR spectra of mixture (25R)-5α-hydroxy-Spirostan-3β,6β-yl phenylcarbamate (**5**).

^{1}H-NMR

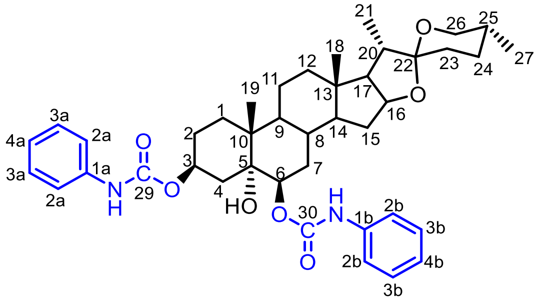

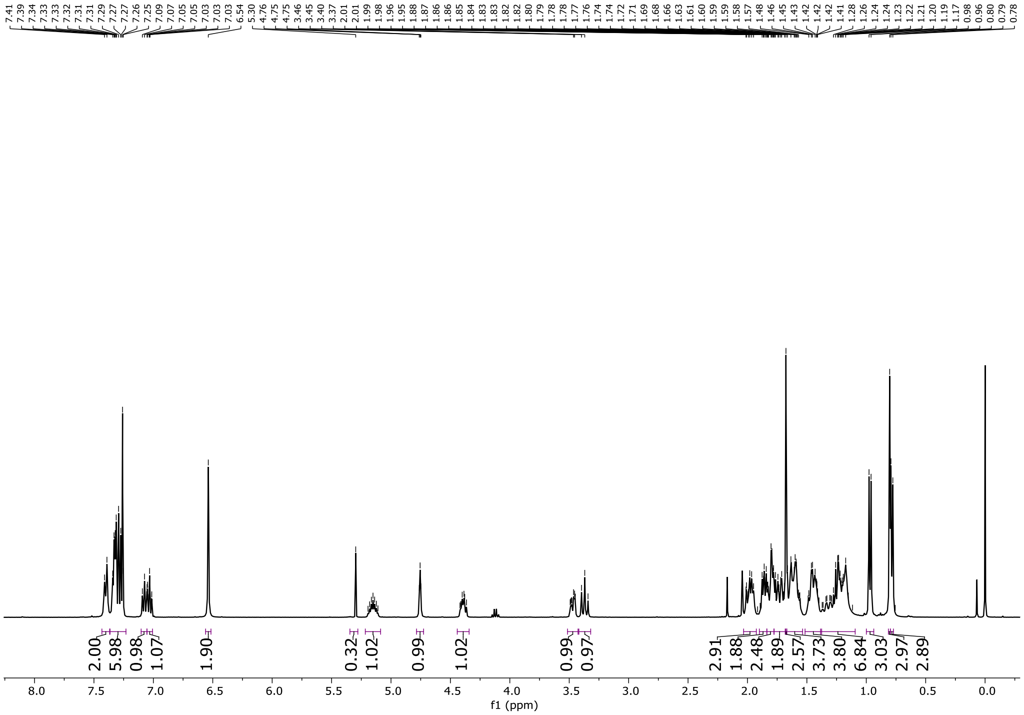

^{13}C-NMR

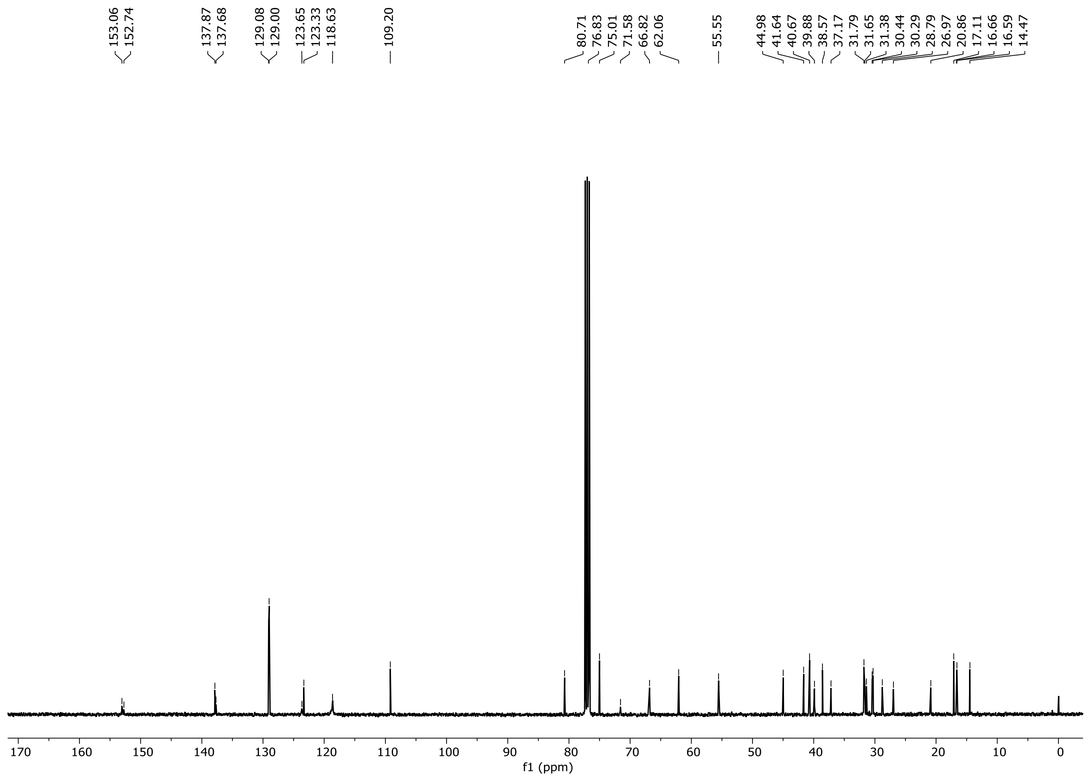

Figure S2: NMR spectra of (25R)- 5α-hydroxy-6-oxo-spirostan-3β-yl phenylcarbamate (**6**).

^{1}H- NMR

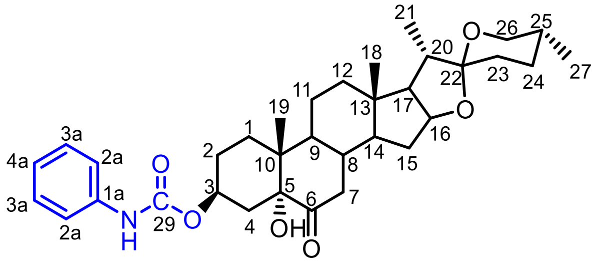

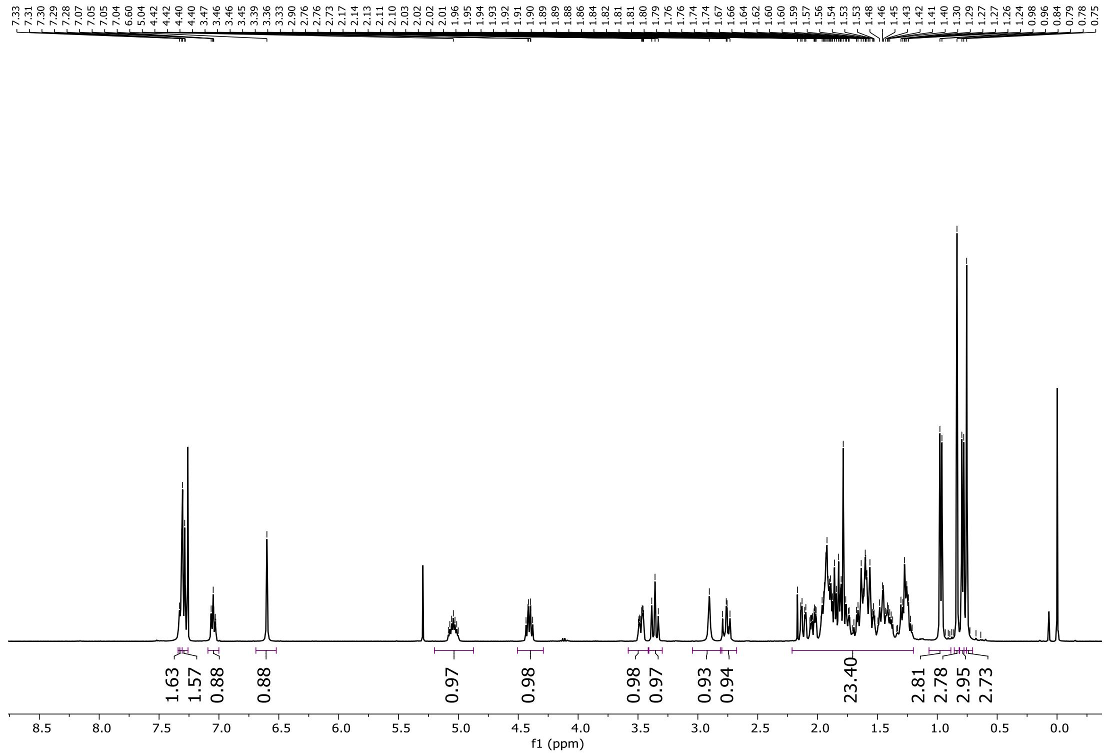
7.33 7.31 7.30 7.29 7.28 7.07 7.05 7.04 6.60 5.04 4.42 4.42 4.40 4.40 3.47 3.46 3.46 3.45 3.39 3.36 3.33 2.90 2.76 2.76 2.73 2.17 2.14 2.13 2.11 2.10 2.03 2.02 2.02 2.01 1.96 1.95 1.94 1.93 1.92 1.91 1.90 1.89 1.89 1.88 1.86 1.84 1.84 1.82 1.81 1.81 1.80 1.79 1.76 1.76 1.74 1.74 1.67 1.66 1.64 1.62 1.60 1.60 1.59 1.57 1.56 1.54 1.53 1.53 1.48 1.46 1.45 1.43 1.42 1.41 1.40 1.30 1.29 1.27 1.27 1.26 1.24 0.98 0.96 0.84 0.79 0.78 0.75
1.63
1.57
0.88
0.88
0.97
0.98
0.98
0.97
0.93
0.94
23.40
2.81
2.78
2.95
2.73
f1 (ppm)

^{13}C-NMR

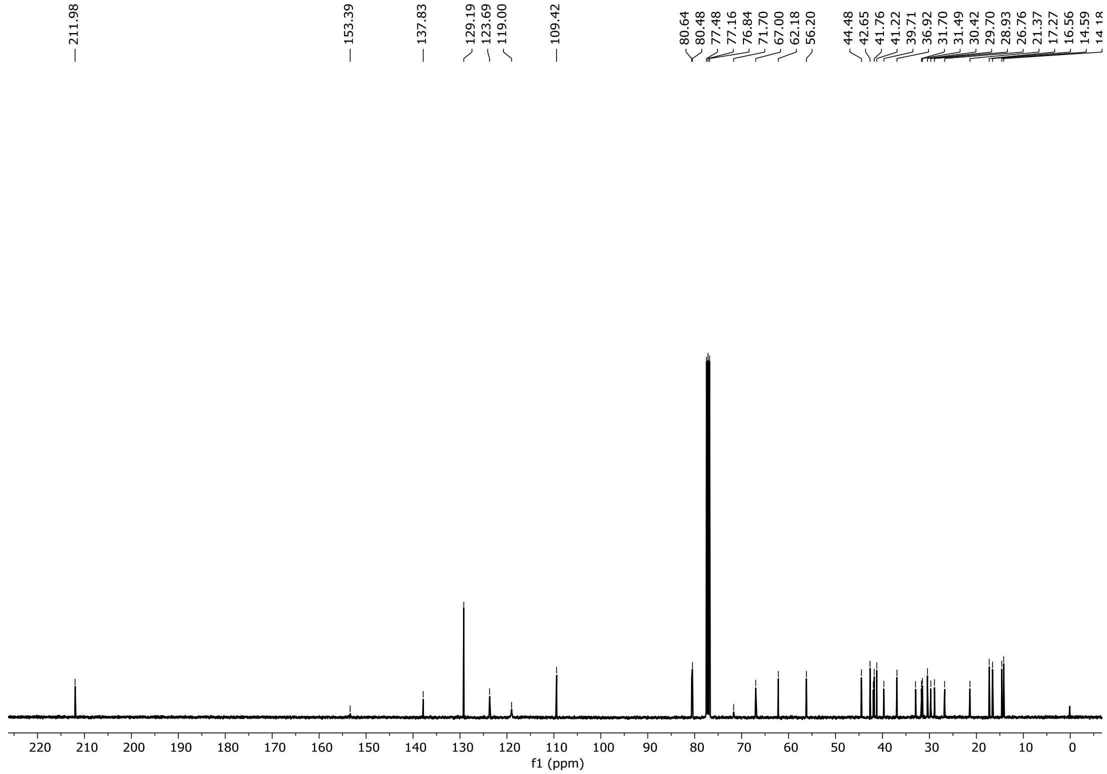
211.98
153.39
137.83
129.19
123.69
119.00
109.42
80.64
80.48
77.48
77.16
76.84
71.70
67.00
62.18
56.20
44.48
42.65
41.76
41.22
39.71
36.92
31.70
31.49
30.42
29.70
28.93
26.76
21.37
17.27
16.56
14.59
f1 (ppm)

Figure S3: NMR spectra of 4-en-androst-17β-yl phenylcarbamate (**7**).

^{1}H- NMR

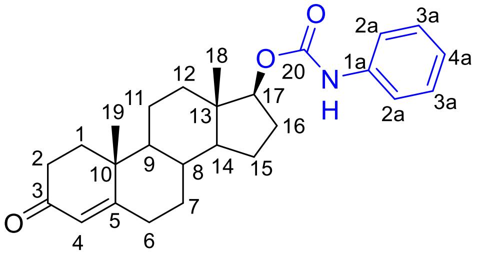

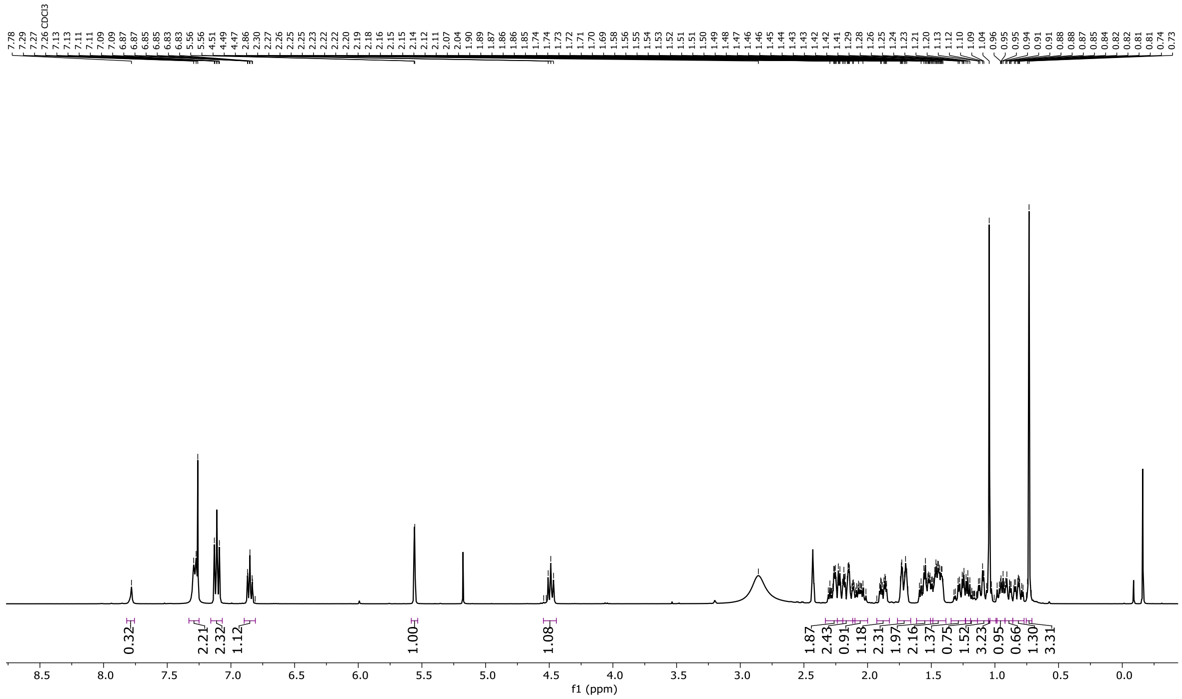

^{13}C-NMR

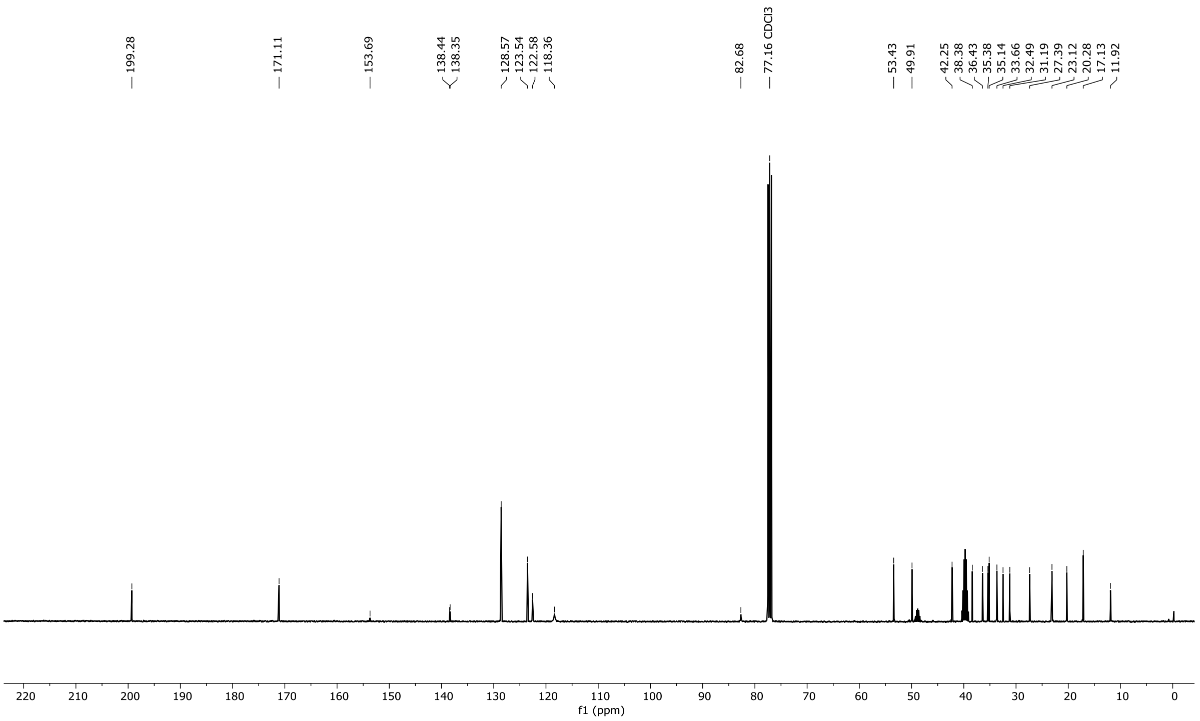
199.28
171.11
153.69
138.44
138.35
128.57
123.54
122.58
118.36
82.68
77.16 CDCl3
53.43
49.91
42.25
38.38
36.43
35.38
35.14
33.66
32.49
31.19
27.39
23.12
20.28
17.13
11.92
220 210 200 190 180 170 160 150 140 130 120 110 100 90 80 70 60 50 40 30 20 10 0
f1 (ppm)

Table S1: Receptor-ligand interactions found by BINANA.

Ligand	Group	Hydrogen Bond	Van der Waals	Short distance interactions	# Similar Interactions	Structure
**4**	1	-	ILE(23), PRO(24), MET(27), VAL(75), THR(76), TYR(80), LEU(99), TRP(169), GLN(313), ARG(316), ILE(317), SER(319)		7	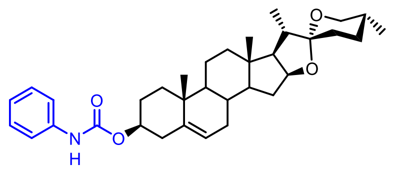
	2	THR(76)	ILE(23), MET(27), VAL(72), THR(76), TYR(80), LEU(99), TRP(169), GLN(313), ILE(315), ARG(316), ILE(317), VAL(320)		8	
**5**	1		PRO(24), THR(76), TYR(80), ARG(316), VAL(320)		5	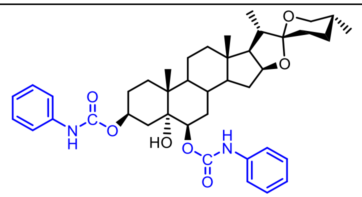
	2		THR(76), TYR(80), ARG(316), VAL(320)	ARG(316) 2.4 Å	4	
**6**	1	TYR (80)	ILE(23), MET(27), VAL(72), VAL(75), THR(76), TYR (80), LEU(99), ILE(315), ARG(316), SER(319), VAL(320)	ARG(316) 2.2 and 2.4 Å	8	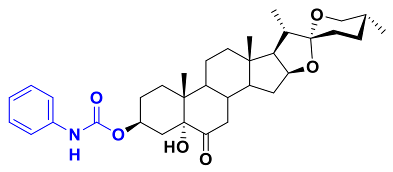
	2	-	ILE(23), MET(27), VAL(72), THR(76),		5	

			GLN(313), ARG(316), ILE(317), SER(319), VAL(320)			
**7**	1	THR(76)	ILE(23), MET(27), VAL(75), THR(76), TYR(80), LEU(99), TRP(169), ILE(315), ARG(316), SER(319), VAL(320).		8	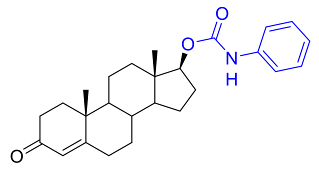
	2	-	ILE(23), PRO(24), MET(27), THR(69), THR(76), TYR(80), ARG(316), SER(319), VAL(320), PRO(322)		6	

Figure S4: Interaction pattern of A) 4(1), B) 4(2), C) 5(1), D) 5(2) E) 6(1), F) 6(2), G) 7(1) and H) 7(3) within the active site of EP4. Hydrogen bonds are highlighted with yellow dashed lines. Residues with Van der Waals interactions are displayed as green sticks. All distances are given in Å.

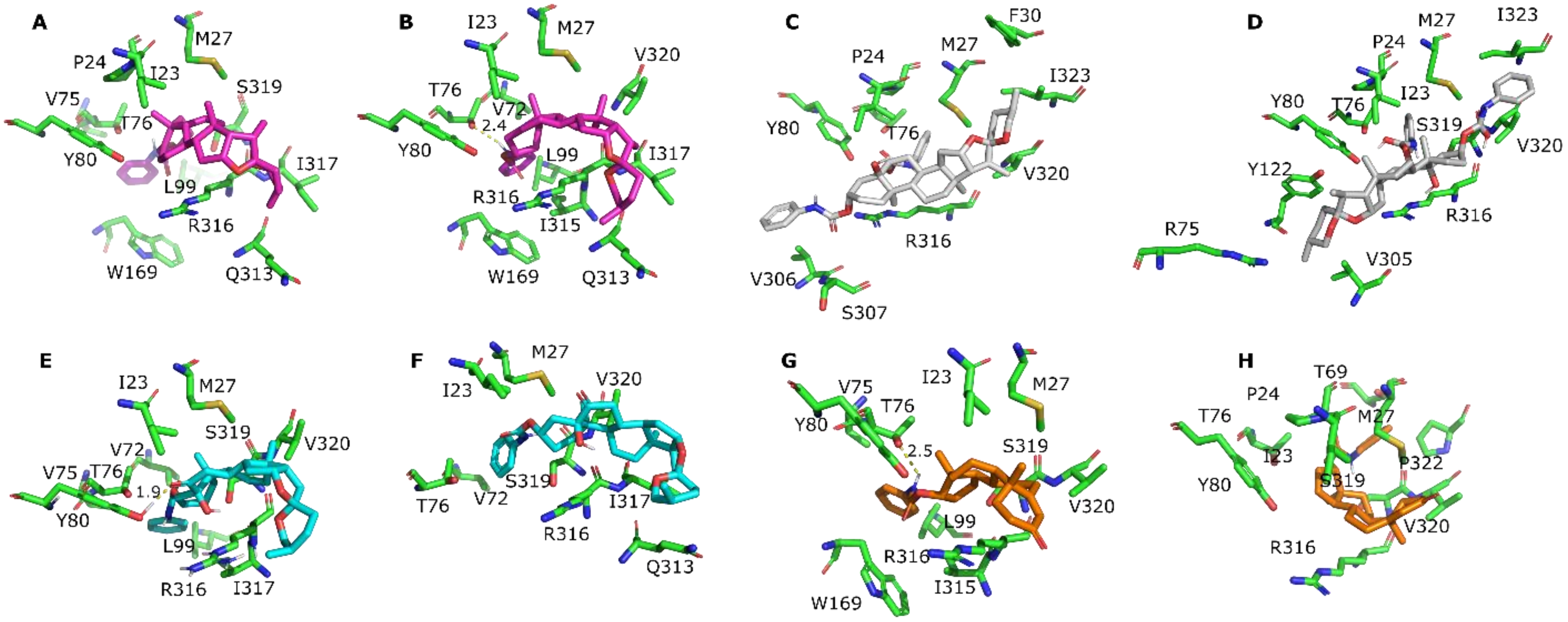

Table S2: Abbreviations. The following abbreviations are used in this manuscript.

h	hours
J	coupling constants
δ	chemical shifts
s	singlet
d	doublet
t	triplet
q	quartet
dt	doublet triplet
dq	doublet quartet
tt	triple triplet
m	multiplet
ax	axial
eq	equatorial
PhNCO	phenyl isocyanate
ppm	parts per millions
rt.	room temperature
TLC	thin-layer chromatography
MTT	[3-(4,5-dimethylthiazol-2-yl)-2,5-diphenyltetrazolium bromide]
